# Regulation and function of the HPV16 CircE7 RNA

**DOI:** 10.1128/jvi.01284-26

**Published:** 2026-09-17

**Authors:** Eunice E. Lee, Yihui Huang, Eleanor Dowell, Kaitlyn Walsh, Calvin Hosler, Austin Witt, Odera Ifeacho, Doreen Palsgrove, Andrew Day, Claire James, Richard C. Wang

**Affiliations:** 1 Department of Dermatology, University of Texas Southwestern Medical Center (UTSW) 12334 , Dallas, Texas, USA; 2 Division of Hematology-Oncology, Department of Internal Medicine, Chang Gung Memorial Hospital Linkou 38014, Linkou, Taoyuan, Taiwan; 3 Medical School, UTSW 12334 , Dallas, Texas, USA; 4 Department of Dermatology, University of Colorado Anschutz Medical Campus (CU AMC), School of Medicine 129263, Aurora, Colorado, USA; 5 Medical School, Louisiana State University Health Sciences Center 12258, New Orleans, Louisiana, USA; 6 Oral and Craniofacial Molecular Biology, Virginia Commonwealth University 6889, Richmond, Virginia, USA; 7 Department of Pathology, UTSW 12334 , Dallas, Texas, USA; 8 Department of Otolaryngology, UTSW 12334 , Dallas, Texas, USA; 9 University of Colorado Cancer Center 171712, Aurora, Colorado, USA; Tufts University, Boston, Massachusetts, USA

**Keywords:** head and neck cancer, RNA splicing, circular RNA, m6A, carcinogenesis, internal ribosome entry site, papillomavirus, translation, transformation

## Abstract

High-risk human papillomaviruses (HPV), including HPV16, produce circular RNA that encompasses the E7 oncogene (circE7). CircE7 can be detected in HPV16-positive cells and tumors, is preferentially localized to the cytoplasm, is N^6^-methyladenosine (m^6^A)-modified, and can be translated into the E7 oncoprotein. Here, we explored the regulation and function of circE7. Mutation of m^6^A motifs flanking the backsplice junction revealed a single m^6^A motif to be essential for circE7 formation. Mutation of this m^6^A motif promoted linear splicing of the E6*I splice site (226^409), suggesting that linear and circular E7 splicing are inversely regulated. Additionally, mutation of an IRES-like motif in circE7 significantly decreased E7 protein expression, without having significant effects on circE7 RNA levels. Knockdown of YTHDC1, but not other m^6^A-binding proteins, decreased both circE7 RNA and protein expression. BaseScope ISH was used to confirm the expression of circE7 in head and neck squamous cell carcinoma cell lines and tumors. Using both qRT-PCR and BaseScope ISH, we found that serum and amino acid starvation significantly increased circE7. Finally, we generated an HPV16 genome with mutations in the circE7 m^6^A motif (Mut2). Stable transduction of primary keratinocytes with Mut2 confirmed the loss of circE7 and increased expression of E6*I. The Mut2 HPV16 genome exhibited significantly decreased viral replication but an increased ability to transform primary keratinocytes. Our studies reveal that the precise regulation of circE7 and E6*I by m^6^A is critical for the ability of HPV16 to infect and transform keratinocytes.

**IMPORTANCE:**

High-risk human papillomaviruses (HPVs), such as HPV16, must carefully control how much E6 and E7 proteins they make. This study shows that HPV16 toggles a single chemical tag on the viral RNA (an m⁶A mark) to control the production of early region RNAs, including a circular RNA called circE7. The same site coordinately regulates splicing of the E6*I isoform. CircE7 uses m⁶A-binding proteins to control its production and a specific sequence to promote its translation. It is present in HPV-positive cancers and can respond to nutrient starvation. Regulation of circE7 through this m^6^A site also impacted viral replication and transformation capacity, indicating that this regulatory mechanism is critical for HPV biology.

## INTRODUCTION

Human papillomavirus (HPV) is a highly prevalent oncogenic virus responsible for up to 5% of all cancers, including up to 80% of oropharyngeal cancers (1, 2). The oncogenic properties of HPV are largely mediated by the early region of high-risk HPV, especially the E6 and E7 oncogenes (3). E6 and E7 work in concert to promote cell-cycle progression through S phase, which enables HPV viral genome replication, while suppressing p53 to block cell cycle arrest and apoptosis. E7 binds to retinoblastoma protein (Rb), inhibiting it and facilitating its degradation, thereby promoting entry into S phase (4). The inappropriate entry of cells into S phase can activate cell cycle checkpoints, which stabilize and increase the levels and activity of the tumor suppressor p53 (3). In high-risk HPV, the full-length E6 protein binds to p53 to inhibit its transcriptional activity and promote its degradation (5, 6). However, excess E6 expression has been shown to induce cellular stress responses and inhibit viral replication (7–9). In high-risk HPV, alternative splicing of the early region can generate a shortened E6*I isoform, which can inhibit full-length E6 abundance and activity. The regulation of alternative splicing in the early region of HPV is a complex, yet critical, aspect of HPV replication and its ability to transform epithelial cells, whose regulation remains incompletely understood.

Interestingly, some viruses have been reported to generate not only linearly spliced RNA but also circular RNAs (circRNA) (10–13). Circular RNAs are produced via backsplicing of a downstream splice donor to an upstream splice acceptor during pre-mRNA splicing (14). Their covalently closed structure lacks the 5′ cap or 3′ poly-A tail of linear mRNA and provides circRNAs higher stability than their linear mRNA counterparts. CircRNAs have been shown to perform a number of biological functions, including regulating miRNA, modulating RNA-binding proteins, and serving as templates for protein translation.

Previously, we demonstrated that the early region of high-risk HPV, including HPV16, produces a circular RNA, circE7, which contains the E7 open reading frame (15, 16). CircE7 is detectable in multiple HPV+ cell lines and primary tumors by transcriptomic sequencing data, RT-qPCR, and Northern blot after RNase R digestion of linear RNA (17, 18). Knockdown of circE7, but not linear E7 isoforms, decreases cancer cell growth *in vitro* and *in vivo*, which can be rescued by expression of circE7. Finally, we provided evidence that circE7 can be translated into E7 protein based on its association with polysomes in an ATG-dependent manner and decreased E7 protein levels after knockdown of circE7. Notably, circE7 is N^6^-methyladenosine (m^6^A)-modified, and this modification is critical for the production of circE7, but not linear E7 isoforms.

M^6^A is a common RNA modification that influences stability, splicing, nuclear export, and translation. Several m^6^A-binding proteins have been implicated in the regulation of circRNAs. Specifically, the m^6^A reader, YTHDF3, has been reported to promote translation of some endogenous circRNAs by recruiting eIF4G2 to m^6^A-modified circRNAs to initiate cap-independent translation (19). YTHDF3 coordinates with other YTH-family m^6^A readers, including YTHDF1, which promotes translation, and YTHDF2, which enhances the degradation of m^6^A-RNAs (20). Additionally, YTHDC1 is a nuclear m^6^A reader that influences RNA splicing and nuclear export and has been shown to be required for backsplicing of circRNAs (21–23). However, the roles of specific m^6^A-binding proteins in the regulation of HPV-derived circRNAs have not been delineated.

In this study, we further characterize circE7. We identify a singular m^6^A motif that regulates the production of circE7 versus linear E6*I. Translation of circE7 is IRES- and YTHDC1-dependent. CircE7 was able to be visualized in HPV16-positive cell lines and primary tissues and exhibits features of dynamic biological regulation. Transfection of cells with a circE7-deficient HPV genome resulted in increased E6*I RNA levels, decreased viral replication, increased cellular transformation, and elevated levels of p53 protein.

## RESULTS

### Discrete m^6^A motif acts as a switch between circE7 and E6*I

**Fig 1:**
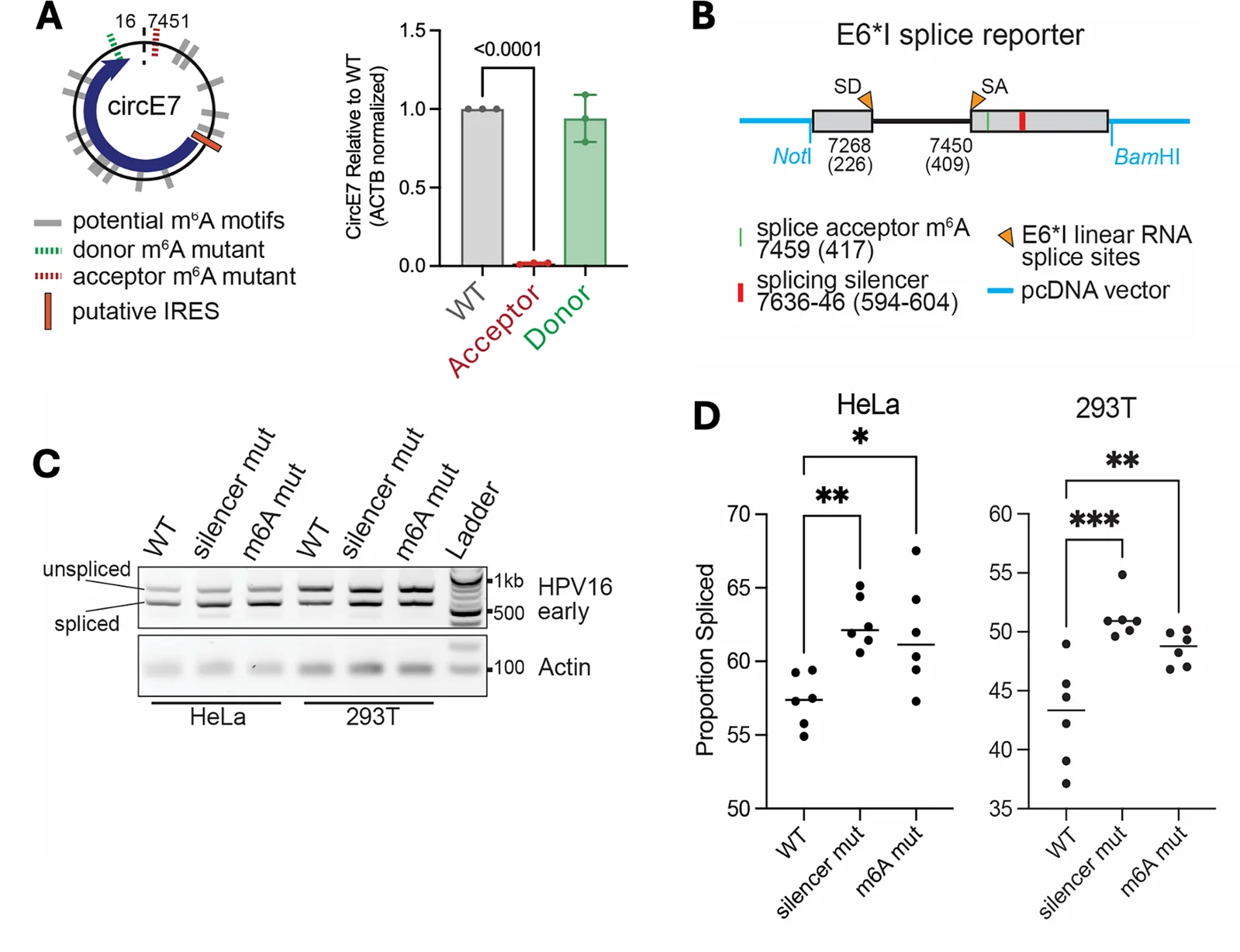
A single m6A site coordinates backsplicing of circE7 and linear splicing of E6*I. (**A**) Mutation of the circE7 backsplice acceptor m6A (red), but not the backsplice donor m6A site (green), significantly decreased circE7 abundance in transfected MDA-1483 cells by RT-qPCR. (**B**) Splicing reporter construct from the early region of HPV16 used to assess the effects on splicing of m6A circE7 acceptor and E7-silencer mutant (27). (**C**) Endpoint RT-PCR identifies unspliced and spliced early region RNA from cells transfected with complete (WT), mutated splicing silencer, or mutated m6A motif constructs. Both silencer and m6A acceptor mutants promote a spliced (lower) band compared to the WT sequence. (**D**) Quantification of C, where proportion spliced was calculated as the spliced vs unspliced band ratio. Dots represent individual replicates; horizontal lines represent means. *P < 0.05, **P < 0.01, ***P < 0.001, *t*-test.

In previous work, we demonstrated that m^6^A motifs surrounding the E7 coding region are essential for circE7 formation (15). Here, we tested whether a specific m^6^A motif is required for circE7 formation. Given the established role of splice site-adjacent m^6^A motifs in splicing regulation (22, 24–26), we focused our efforts on the two candidate m^6^A motifs located immediately adjacent to the circE7 backsplice junction. Transversion mutations of the adenosine residue in the m^6^A motifs located near the splice donor (m^6^A-SD-853) and splice acceptor (m^6^A-SA-418) of the circE7 backsplice junction were generated (Fig. 1A). Transfection of these mutant circE7 expression constructs revealed that the splice acceptor motif (m^6^A-SA-418) is essential for circE7 RNA production, while the splice donor motif (m^6^A-SD-853) did not show consistent effects on circE7 RNA levels. These effects were reproducible across several different cell lines (Fig. S1).

To investigate the role of the splicing acceptor m6A motif (m^6^A-SA-418) in linear splicing, we designed a splice reporter plasmid construct containing the previously described E6*I splice site (226^409) but lacking the circE7 backsplice donor (SD880). Two mutant constructs based on this splice reporter plasmid—a splice acceptor m^6^A motif mutant (m^6^A-SA-418) and a previously described splicing silencer mutant (27)—were generated (Fig. 1B). Endpoint RT-PCR from cells transfected with the wild-type reporter construct revealed bands consistent with both spliced and unspliced RNA (Fig. 1C and D). As expected, mutation of the splicing silencer motif resulted in increased linear 226^409 splicing. Notably, the single acceptor motif (m^6^A-SA-418) transversion mutant also increased linear 226^409 splicing. These results suggest that the splice acceptor m^6^A motif coordinately suppresses linear 226^409 (E6*I) splicing while promoting circE7 formation.

### Regulation of circE7 translation

**Fig 2:**
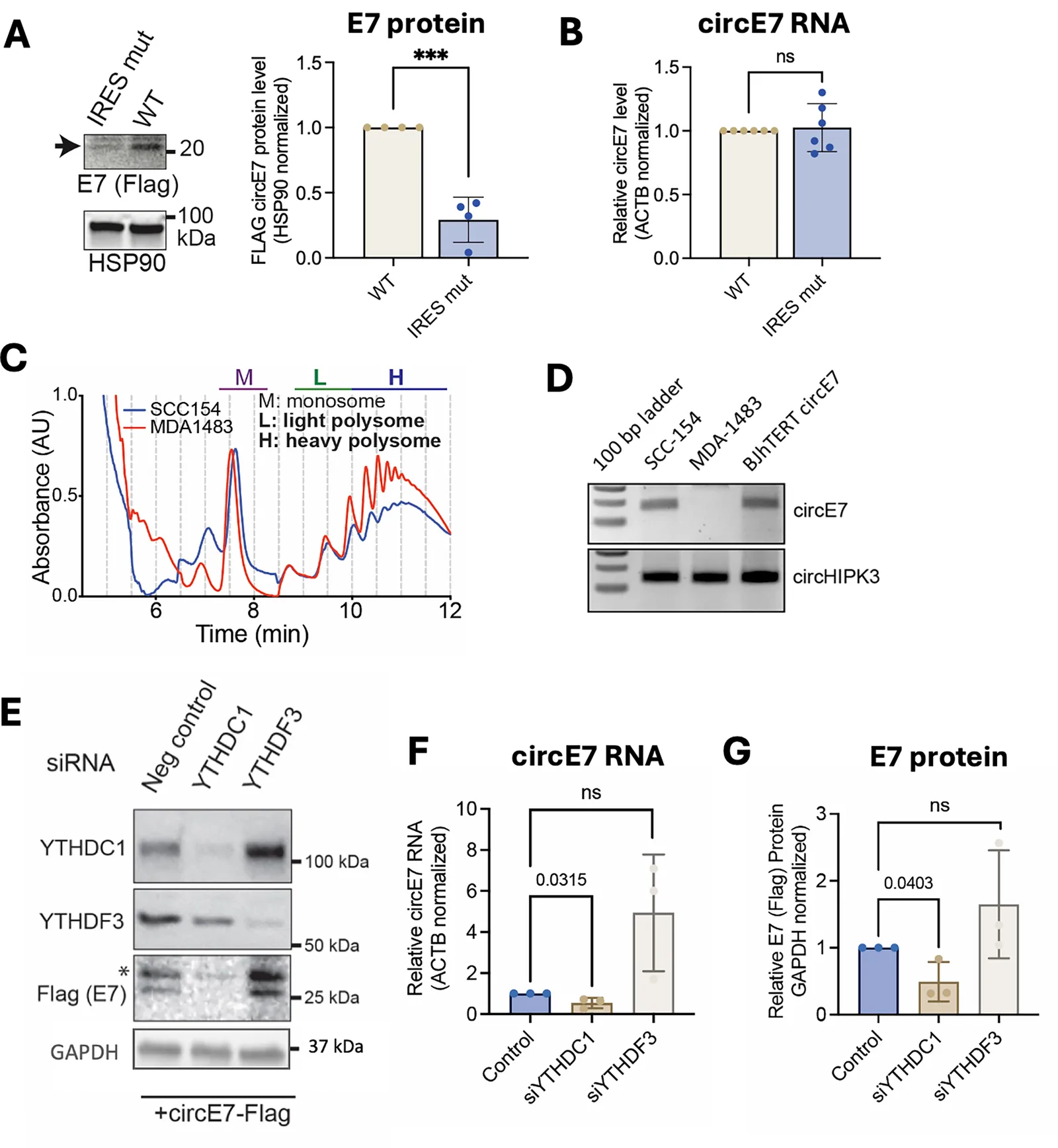
CircE7 RNA translation is dependent on an IRES-like motif and m6A-binding proteins. (**A**) Western blot from MDA-1483 cells transfected with WT or IRES mut circE7 plasmid. Representative blot (left), quantification (right). ***P < 0.001. (**B**) RNA levels of the IRES mutant circE7 RNA by RT-qPCR in MDA-1483 cells. Difference not statistically significant by *t*-test. Twice as much RNA was transfected to get equivalent levels of RNA between IRES and WT. (**C**) Representative tracing of circE7-transfected cells after polysome enrichment assay with the monosome (M), light polysome (L), and heavy polysome (H) fractions indicated. Polysomes isolated from SCC-154 HPV+ (blue) and MDA1483 HPV− (red) cells. (**D**) Endpoint PCR of CircE7 or circHPK3 in light and heavy polysomes from SCC-154 (HPV+) cells, MDA1483 (HPV−) cells, and BJ cells transfected with hTERT circE7. All samples were RNase R-treated. (**E**) Western blot from HeLa cells co-transfected with a circE7-Flag expression vector and the indicated siRNA. HSP90, loading control. *indicates non-specific band. (**F**) RT-qPCR for circE7 RNA levels after YTHDC1 and YTHDF3 knockdown (*n* = 3). (**G**) Quantification of western blot (*n* = 3).

CircE7 can be translated to produce E7 protein (15). After transient transfection, circE7 associates with polysomes, suggesting that overexpressed circE7 is translated. To extend these findings, we assessed whether circE7 associates with polysomes at physiological levels. Polysomes were isolated from two squamous cell cancer cell lines, HPV16-positive SCC154 cells and HPV16-negative MDA1483 cells. RT-PCR of polysome fractions from SCC154 cells detected circE7 RNA, while it was not detected on MDA1483 polysomes (Fig. 2A and B).

Previous studies revealed that the early (E6−E7) region of HPV16 contains sequences capable of promoting cap-independent translation, possibly through base pairing and interaction with the 18S ribosomal RNA (28). Genome comparisons between high-risk and low-risk HPV further narrowed a region complementary to 18S to a short region in the 3′ end of E6, which is contained in circE7 (29). To test whether this sequence could function as an IRES-like motif, we generated an IRES-like mutant circE7 in which nucleotides complementary to 18S were substituted with transversion mutations (Fig. 2C). WT or IRES-like mutant Flag-tagged circE7 plasmids were transfected into MDA1483 cells. Strikingly, mutation of the IRES-like mutant circE7 markedly reduced levels of E7 protein, despite comparable circE7 RNA abundance (Fig. 2D). CircRNAs were confirmed to be circular in structure due to resistance to RNase R digestion (Fig. S2). These results indicate that the primary sequence of circE7 is critical for its translation, possibly through IRES-like elements that mediate ribosome recruitment.

### Role of m^6^A-binding proteins in CircE7 formation and translation

Given the critical role of m^6^A sites in circE7 function, we next investigated whether m^6^A-associated RNA binding might also influence circE7 formation. YTHDC1 has been reported to be involved in backsplicing of endogenous circRNA (23, 30). Co-transfection of cells with the Flag-circE7 plasmid and either YTHDC1 or YTHDF3 siRNA resulted in the expected decrease in YTHDC1 or YTHDF3 protein expression, respectively (Fig. 2E). YTHDC1 knockdown significantly decreased circE7 RNA levels (Fig. 2F), accompanied by a marked reduction in E7 protein production (Fig. 2G). In contrast, YTHDF3, which has been reported to interact with eIF4Gs and promote endogenous circRNA translation (19), did inconsistently increase both circE7 RNA and protein levels in MDA-1483 cells; however, this effect was not statistically significant nor was it reproducible in additional cell lines (Fig. 2E through G and Fig. S3A). Other m^6^A-related proteins, including YTHDC2 and hnRNPL, have been implicated in endogenous circRNA formation (31, 32). However, knockdown of neither YTHDC2 nor hnRNPL inhibited circE7 formation (Fig. S3B through D). Surprisingly, knockdown of hnRNPL increased both circE7 RNA and E7 protein levels (Fig. S3B through D), suggesting more complex roles for m^6^A-binding proteins in circE7 formation. In summary, we confirm YTHDC1 plays the key role in circE7 formation, while other m^6^A-related binding proteins appear to exert more nuanced effects.

**Fig 3:**
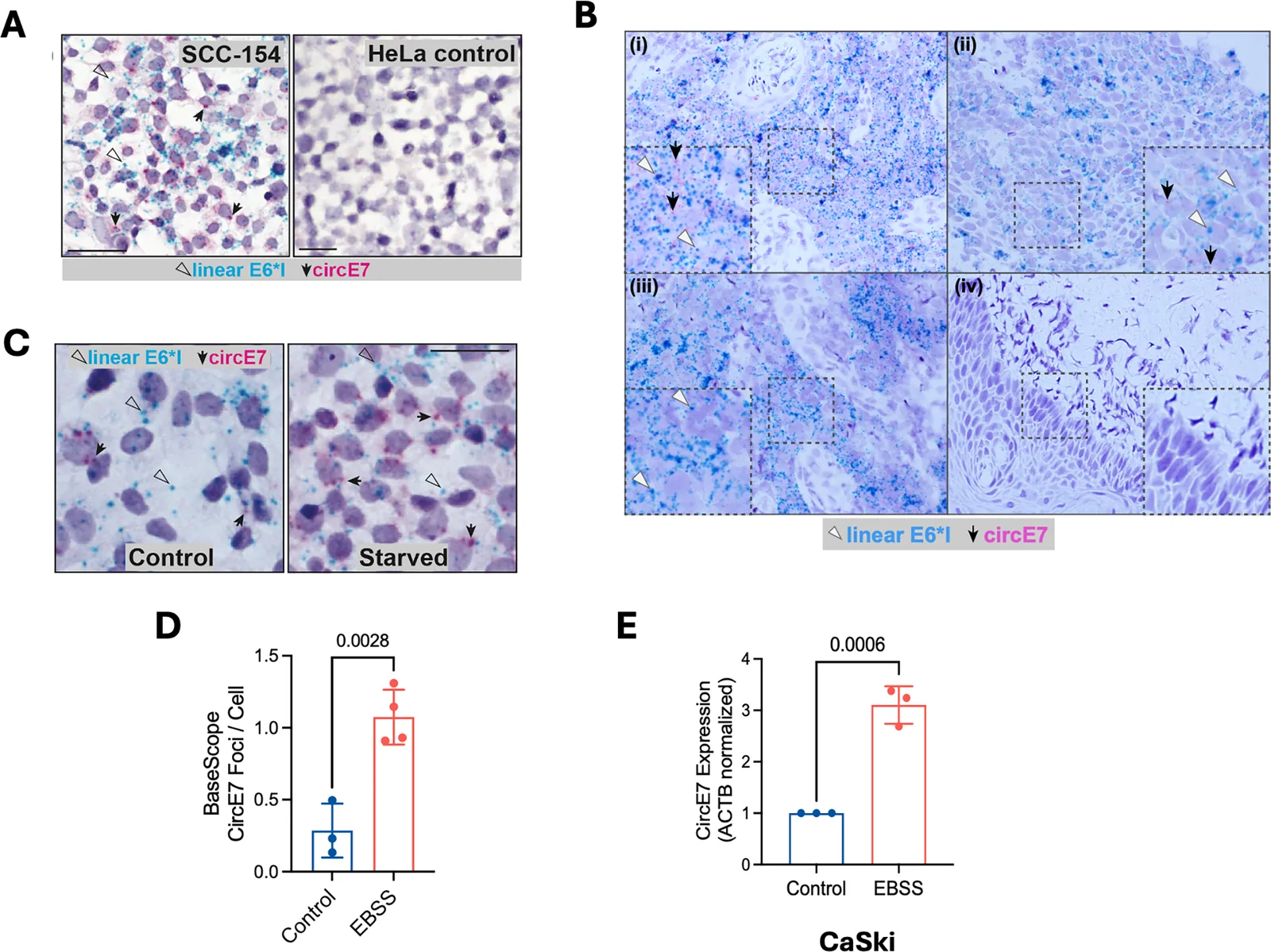
CircE7 RNA is upregulated during cellular starvation. (**A**) SCC-154 (HPV16+) and negative control HeLa cells (HPV16−) were probed by BaseScope Duplex custom probes for either E6*I or circE7. Bar, 50 mm. Arrows highlight the presence of circE7 (red) or E6*I (cyan) probes. (**B**) BaseScope detection of circE7 and linear E6*I in HPV + HNSCC FFPE sections. (i and ii) Visualization of circE7 (red) and linear E6*I (cyan) in p16 + HNC. (iii) Linear E6*I (cyan) but minimal circE7 in a p16 + HNC. (iv) No detection of either circE7 or linear E6*I in HPV− adnexal tumor. (**C**) SCC-154 cells were probed for linear (E6*I) or circE7 RNA by BaseScope with or without starvation in EBSS (4 h). (**D**) Quantification of results shown in panel C. Starvation significantly induced circE7 levels as assessed by BaseScope Quantification of BaseScope foci (right) (*n* = 4, unpaired *t*-test). Bar, 50 mm. (**E**) EBSS starvation induced circE7 levels in SCC-154 (B) and CaSki (C) as assessed by RT-qPCR (*n* = 3, unpaired *t*-test).

### Detection of CircE7 *in vivo* and physiologic regulation of CircE7

While NGS and RT-PCR have confirmed that circE7 is expressed in HPV16+ tumors (15, 17, 18), these methodologies do not allow for the visualization of circE7 *in situ*. To detect circE7 directly in cells *in situ*, we utilized BaseScope, an RNA *in situ* method capable of discriminating between RNA at near-nucleotide resolution (Fig. S4) (33). Using this approach, we confirmed that distinct E6*I and circE7 isoforms are detectable in the HPV16-positive SCC-154, while neither isoform was detected in HPV16-negative HeLa cells (Fig. 3A). Both E6*I and circE7 were also detectable in HNSCC FFPE tissue sections (Fig. 3B). In two cases of HPV16-positive HNSCC, BaseScope analysis showed RNA ISH specific for both circE7 and E6*I (i and ii). In one HPV16-positive HNC tissue section, circE7 was not identified, while E6*I was detected (iii). As expected, neither circE7 nor E6*I was detected in the HPV16-negative tumor control sections (iv) or in microcystic adnexal carcinoma and mucoepidermoid carcinoma (Fig. S5). These tumor-specific differences in the expression of circE7 and linear E6*I suggest complexities in the regulation of E6*I and circE7 splicing.

**Fig 4:**
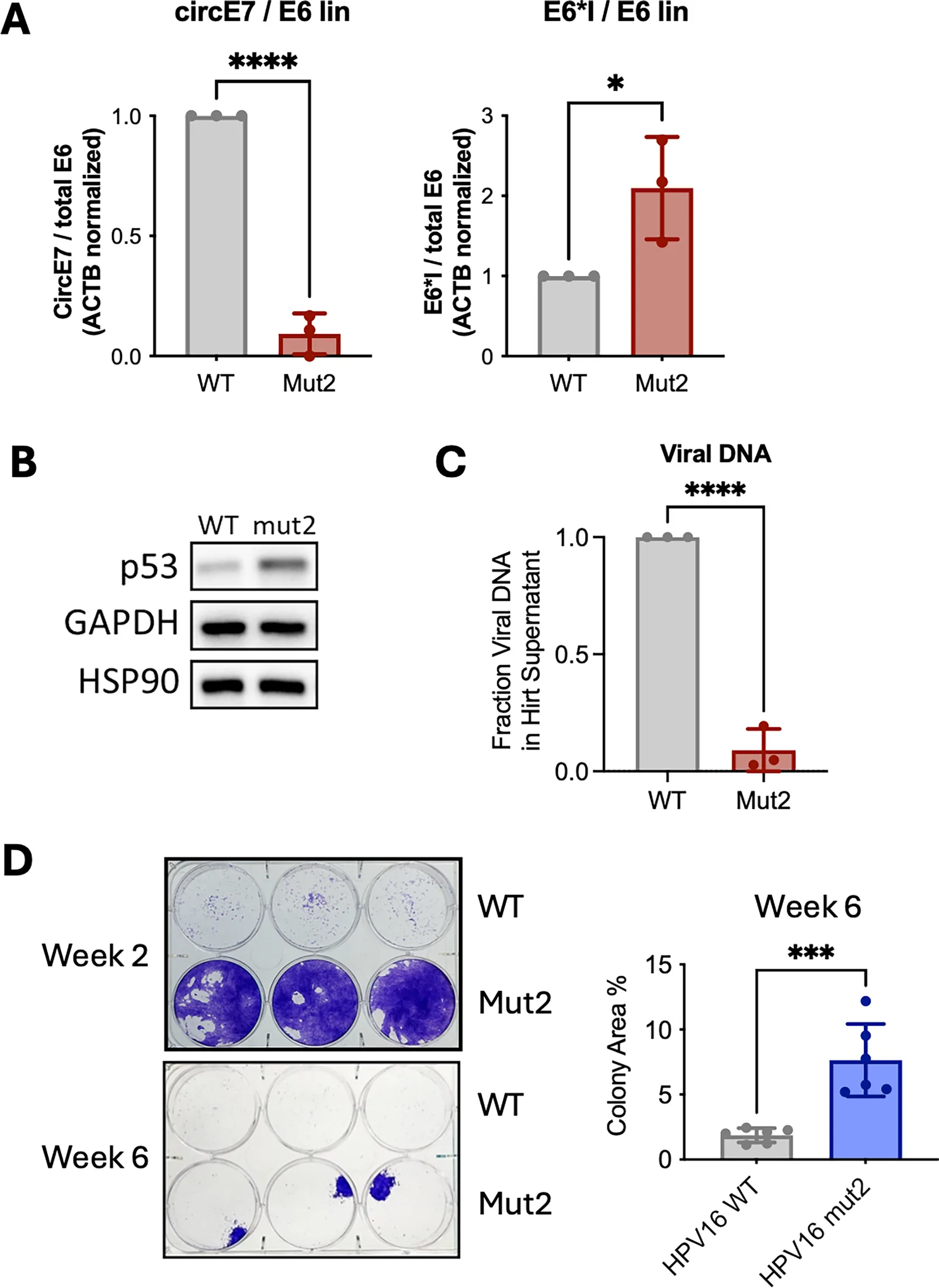
Impact of the circE7 m6A motif on HPV16 replication and immortalization. (**A**) RT-qPCR of circE7 and E6*I normalized to linear E6 RNA from primary keratinocytes transfected with HPV16 WT or HPV16 mut2 genomes (circE7 splicing acceptor m6A motif mutation, no change to AA sequence). *P < 0.05, ****P < 0.0001. (**B**) Western blot of p53, GAPDH, and HSP90 in whole cell extracts from keratinocytes transfected with HPV16 WT or mut2 genomes. (**C**) Viral DNA detected by qPCR after HIRT DNA extraction from primary keratinocytes transfected with either wild-type or mut2 HPV16. ****P < 0.0001. (**D**) Crystal Violet staining of keratinocytes grown in 10% BCS DMEM for 2–6 weeks. Cells were transfected with either wild-type HPV16 or HPV16 Mut2. Representative images (left) and quantification at week 6 (right). ***P < 0.001

In addition to inter-tumor variability in circE7 expression, even circE7+ tumors showed intra-tumoral heterogeneity of circE7 expression. Tumors exhibit heterogeneous microenvironments that impose distinct adaptive demands on cells located in the core versus the periphery (34, 35). Previously, we noted that increased cell density augments circE7 expression (23). Notably, m^6^A modifications have been reported to increase in response to nutrient starvation (36, 37). We therefore tested whether nutrient availability influences circE7 expression by treating SCC154 cells with amino-acid and serum-free Earle’s-buffered salt solution (EBSS) media. BaseScope for E6*I and circE7 showed a marked and significant increase in circE7 levels, with the majority of cells now containing at least one circE7 foci (Fig. 3C and D). To orthogonally validate this finding, we performed RT-qPCR with circE7-specific primers and detected a 2-fold to 3-fold increase in circE7 in starved CaSki cells (Fig. 3E). Additionally, while circE7 levels increase after EBSS starvation, E6*I transcripts decrease (Fig. S6), supporting specific dynamic regulation of circE7. We conclude that nutrient starvation may be one physiological trigger that can modulate circE7 levels.

### Effects of CircE7 on HPV16 genome replication and keratinocyte transformation

To investigate the influence of m^6^A-SA-418 on HPV16 replication, we generated a mutant HPV16 genome in which m^6^A-SA-418 cannot be methylated. Two substitutions were generated, which abolished the m^6^A motif (TAACT–CAATT) without affecting the coding sequence of E6, named Mut2. We transfected wild-type (WT) and Mut2 genomes into primary human keratinocytes using previously established protocols (38). Cells underwent drug selection for 6 days, after which both WT and Mut2-transfected keratinocytes were able to generate stably infected keratinocytes. Sanger sequencing confirmed successful transduction of the expected wild-type and Mut2 HPV16 genomes.

Both RNA and Hirt DNA extracts were collected from early-passage keratinocytes. Using RT-qPCR, we confirmed that circE7 was significantly reduced in Mut2-transfected keratinocytes (Fig. 4A, left). Consistent with our *in vitro* studies, levels of E6*I increased, further supporting the notion that m^6^A-SA-418 acts as a regulatory switch between circE7 and E6*I (Fig. 4A, right). Given the increase in E6*I, we assessed p53 protein levels in Mut2. Western blot revealed that p53 levels were increased in Mut2, consistent with a decrease in E6-mediated degradation of p53 (Fig. 4B). Importantly, E6 transcript levels do not significantly decrease as we would expect if the change in p53 is driven by loss of E6 in the Mut2 background (Fig. S7). Therefore, we conclude that increased E6*I negatively regulates full-length E6 protein function, resulting in increased p53 levels.

We next assessed whether the loss of m^6^A-SA-418 affects viral genome replication. Quantitative PCR of early-passage Hirt extracts revealed that the m^6^A-SA-418 mutant contained approximately 10-fold less viral DNA in Hirt supernatant (Fig. 4C), suggesting significantly impaired viral replication. Finally, we assessed the ability of WT and m^6^A-SA-418 mutant HPV16 to transform primary keratinocytes. No consistent differences were noted in survival after initial selection and immortalization. Next, cultures were transferred from basal keratinocyte media to DMEM supplemented with 10% BCS for up to 6 weeks (Fig. S8). After both 2 and 6 weeks in DMEM, m^6^A-SA-418 transfected keratinocytes showed greater viability compared with WT HPV16-transfected cells as assessed by crystal violet staining. Thus, despite impairments in viral replication, the m^6^A-SA-418 mutant HPV resulted in prolonged survival in 10% BCS, indicating greater resistance to differentiation than wild-type HPV16 genomes (Fig. 4D). To rule out the difference in transformation resulting from differences in viral genome integration between WT and Mut2, we quantified percent integration by ExoV assay (Fig. S9). Both WT and Mut2 genomes were substantially episomal, and there was no significant difference between WT and mut2 integration rates.

## DISCUSSION

This study provides a detailed model of circE7 regulation and function within the context of HPV infection. We demonstrate that the m^6^A-SA-418 motif acts as a critical regulatory switch for circE7 backsplicing and that an IRES-like motif is required for circE7 translation. Knockdown of the m^6^A reader YTHDC1 decreases both circE7 RNA and E7 protein levels, indicating that it is likely an important factor for circE7 backsplicing, while YTHDF3 did not have an impact on circE7 backsplicing or translation. Furthermore, circE7 is physiologically regulated during serum starvation, indicating a dynamic and directed regulation of circE7. Using a splicing reporter, we demonstrate that circE7 backsplicing and E6*I forward splicing are inversely regulated. In the context of the HPV genome, a circE7-backsplicing-defective mutant (Mut2) demonstrates increased relative levels of E6*I transcript and elevated p53, consistent with the established role for E6*I in inhibiting E6 activity. Cells infected with Mut2 showed impaired viral genomic replication but increased transformation. The ability of circE7 to inhibit transformation is also consistent with our previous studies suggesting that high levels of circE7 correlated with an improved prognosis in HPV+ cervical, head and neck, and anal squamous cell cancers (15, 17, 18). Together, this paints the picture that circE7 is critical for many aspects of HPV function and could act as a critical regulatory node to modulate HPV early region activity in response to cellular conditions.

This study contributes to the growing body of literature that a subset of circRNAs are translated into protein. Previously, we demonstrated that overexpressed circE7 associates with polysomes in an ATG-dependent manner and that shRNA specifically targeting the circE7 backsplice junction reduces E7 protein levels, an effect rescued by shRNA-resistant circE7 (15). Here, we build on these observations by demonstrating that circE7 translation is promoted by an IRES-like sequence, and that mutation of this sequence significantly reduces E7 protein production. Additionally, we show that circE7 associates with polysomes in HPV16+ cell lines at endogenous levels, supporting that circE7 is translated in native contexts.

In this study, we identified that the m^6^A motif, m^6^A-SA-418, regulates circE7 production. The backsplicing of other circRNAs, such as circZNF609, has been shown to be regulated through m^6^A residues (23). This is the first study to show circRNA backsplicing to be regulated by a single m^6^A site. Several prior studies have identified a role for the m^6^A reader, YTHDC1, in circRNA formation (23, 30, 39). Our data support a similar role in circE7 biogenesis, although our studies do not exclude the participation of additional m^6^A readers in circE7 formation. While the m^6^A reader YTHDF3 has been shown to be important for the translation of human genome-encoded circRNA (19, 23), knockdown of YTHDF3 did not consistently reduce circE7 translation, indicating that YTHDF3 is not universally essential for circRNA translation. Instead, our data support that circE7 translation is mediated by an IRES-like sequence, highlighting that, like their linear coding RNA counterparts, circRNAs may be translated via multiple mechanisms. M^6^A modifications have been reported to change dynamically in response to cell stressors, including nutrient starvation (36). Similarly, circE7 levels increased in response to starvation and high cell density (16), suggesting that circE7, through its m^6^A motif and YTHDC1, is dynamically regulated and deployed in response to changing cellular conditions. Emerging literature suggests that m^6^A modification plays complex roles in the regulation of HPV splicing. Specifically, Cui et al. showed that globally perturbing m^6^A writers, readers, and erasers could influence HPV16 splicing (40). Specifically, overexpression of the demethylase ALKBH5 promoted E6 intron retention, and overexpression of m^6^A reader YTHDC1 also promoted intron retention and full-length E6 mRNA expression, indicating that m^6^A motifs play a complex role in HPV splicing regulation. In addition, it has been suggested that multiple m^6^A-modified sites in HPV18 influenced HPV18 RNA splicing (41). Notably, they show that m^6^A methylation of position 455 in HPV18 correlated with unspliced linear RNA. This motif is in a similar relative position to m^6^A-SA-418 in HPV16 and could perform similar splicing regulation functions. Our work provides evidence that a single, specific m^6^A modification regulates not only linear E6 splicing but also circE7 backsplicing.

Our data support a model in which the single m^6^A site, m^6^A-SA-418, acts as a master toggle between circE7 and linear E6*I. Loss of circE7 correlated with an increase in E6*I RNA. This alternative splicing balance allows HPV to finely tune E6*I and full-length E6 to control E6 activity, while maintaining E7 protein levels, as E7 can be translated from both E6*I and circE7. Such nuanced regulatory mechanisms are critical for HPV infection, given the compact genome and shared promoters for all early genes (7). Our findings contribute to our understanding of E6 and E7 splicing, adding to previously described regulators of early region splicing (27, 38, 42). Post-translational regulation of E6, E7, and their interacting partners adds an additional layer of complexity to allow for the precise tuning of E6 and E7 function in facilitating viral replication (43).

Several genetic features distinguish low-risk HPV from high-risk HPV in terms of transforming potential, including the differential abilities of E6 and E7 proteins to engage p53 and Rb, respectively, and the presence of a C-terminal PDZ-binding motif in high-risk HPV (44, 45). Similarly, circE7 RNA isoforms have also only been detectable in high-risk HPV, raising the possibility that evolution of the high-risk HPV early region and backsplicing are linked. We conclude that regulation of circE7 via m^6^A-SA-418 allows HPV to tune early genes E6 and E7 levels to distinctly modulate individual protein expression from a single pre-mRNA. Additional studies of this densely encoded and tightly regulated genomic region will continue to yield insights into both viral and human RNA biology.

## MATERIALS AND METHODS

### Cell culture

Primary human keratinocytes were purchased from ATCC (PCS-200-010) and cultured in Dermal Cell Basal Medium (ATCC, PCS-200-030) supplemented with Keratinocyte Growth Kit (PCS-200-040) on Collagen I-coated plates. SCC-154 cells (ATCC, CRL-3241) were cultured in DMEM/F-12 GlutaMAX media (Gibco, 10565018) supplemented with 10% FBS, 0.1 µg/mL hydrocortisone (Sigma, H0888), and 10 ng/mL EGF (Gibco, PHG0311L). HeLa (ATCC, CCL-2), 293T (Takara, 632180), and MDA-1483 (gift from CM Chiang lab) were cultured in DMEM with 10% FBS. CaSki cells (ATCC, CRM-CRL-1550) were grown in RPMI with 10% FBS.

### EBSS starvation experiments

For EBSS starvation experiments, cells were seeded in 6-cm dishes and incubated overnight in standard growth media. On the following day, cells were washed twice with EBSS to remove traces of media and then incubated in EBSS for 4 h at 37°C. Total RNA was isolated using the RNeasy Mini Kit (Qiagen, 74106), and 400 ng of RNA was used for cDNA synthesis using the Superscript IV Synthesis Kit (Invitrogen, 18091050). For RT-qPCR analysis, 2 µL of diluted cDNA (1:10 in water) was used in each reaction.

### RNA isolation and cDNA synthesis

RNA was isolated from cells using the RNeasy Mini Kit (Qiagen, 74106) according to the manufacturer’s instructions. To digest linear RNA, 2.5 U RNase R (Lucigen, RNR07250), 0.25 U DNase (Qiagen, 79254), 5 U murine ribonuclease inhibitor (New England Biolabs, M0314S), and 1× RNase R reaction buffer were added to each microgram of total RNA and incubated at 37°C for 45 min. Reactions were then placed on ice, combined with 1 µL of 1 mM EDTA and 1 µL random hexamers (100 µM), and denatured at 65°C for 5 min. Samples were then reverse-transcribed using the Superscript IV Synthesis Kit (Invitrogen, 18091050) according to the manufacturer’s protocol. For standard reactions without RNase R digestion, 1 µg of total RNA was used for cDNA synthesis unless otherwise stated. RNase R digestion was confirmed by qPCR for linear RNAs using the PowerUp SYBR Green Master Mix (Applied Biosystems, A25742).

### Endpoint PCR and RT-qPCR

Endpoint PCR was performed using the SapphireAmp PCR Master Mix (Takara, RR350B) as previously described (46). For endpoint PCR and qRT-PCR, cDNA products were diluted 1:10 in water, and 2 µL of the diluted product was used in each reaction. Real-time PCR analysis was performed using PowerUP SYBR Green Master Mix (Applied Biosystems, A25742) according to the manufacturer’s instructions, and data were normalized using ß-actin as a loading control. To specifically detect full-length E6 RNA, primers were designed with one primer between HPV genomic positions 226 and 409, which is spliced out in E6*I and other truncated E6 isoforms. See Table S2 for all primer sequences.

### Western blot

For western blot analysis, cells were lysed in Cell Lysis Buffer (Cell Signaling Technologies, 5872S), separated on SDS-PAGE gels, and transferred to PVDF membranes. Membranes were incubated overnight at 4°C with the following primary antibodies: YTHDC1 (Cell Signaling, 77422), YTHDF3 (Cell Signaling, 24206), YTHDC2 (Cell Signaling, 46324), FLAG (Sigma, A8592), GAPDH (Santa Cruz, sc-32233), and HSP90 (Cell Signaling, 4877S). Western blots were then incubated with the appropriate HRP-conjugated secondary antibodies (Cell Signaling, 7076S and 7074S) and ECL substrate (Perkin Elmer, 50-904-9323) for signal detection.

### Polysome purification and RNA preparation

Polysome fractionation and analysis were performed as previously described (Zhao, 2019). Briefly, SCC-154 and MDA-1483 cells were grown to 60%–80% confluence in 15-cm dishes and lysed in polysome lysis buffer (20 mM Tris pH 7.4, 5 mM MgCl_2_, 100 mM NaCl, 0.1% NP-40, and 100 µg/mL cycloheximide) in the presence of EDTA-free protease cocktail (Roche, 11836170001). To pellet nuclei, cell lysates were centrifuged at 12,000 × *g* for 10 min at 4°C, and the supernatant was transferred onto 10%–50% (wt/vol) sucrose gradient columns and ultracentrifuged at 20,000 × *g* for 2 h at 4°C. RNA was extracted from collected fractions using TRIzol LS Reagent (Invitrogen, 10296010) according to the manufacturer’s instructions. After isolation, 650 ng of RNA was first RNase R-digested and then used as a template for random hexamer-primed cDNA synthesis (Invitrogen, 18091050). cDNA products were diluted 1:10 in water for endpoint PCR reactions.

### E6*I splice reporter

A synthetic DNA containing the E6-E7 early region (NC_001526, nt 7140-7900, and 16ER_splice_Not_Bam) was cloned into pcDNA3.1(−) as a NotI, BamHI fragment to generate the E6*I splice reporter construct. Subsequently, synthetic DNAs containing transversion mutations in the m^6^A motif (16ER_m^6^A_Not_Age) or a previously described splicing silencer (16ER_silence_mut_Age_Bam) were cloned as NotI, AgeI or AgeI, and BamHI fragments, respectively, into the wild-type splice reporter construct. Splice reporter constructs were transfected into the indicated cell lines (293T, HeLa), and total RNA was isolated (Qiagen, mini RNA-easy). Endpoint RT-PCR was conducted to evaluate the E6*I splice region, and bands corresponding to the unspliced and spliced RNA were identified. Bands were quantified with ImageJ as a proportion of total RNA.

### IRES mutant plasmid

Primer-mediated mutagenesis was conducted on pcDNA3.1(−) circE7_WT and pcDNA3.1(−) circE7_Flag1 (15, 16) using the “CircE7_IRESmut_F” and “CircE7_IRESmut_R” primers with the NEB Q5 Site-Directed Mutagenesis kit according to the manufacturer’s instructions. Sequences were confirmed by Sanger sequencing.

### siRNA knockdown

One day prior to transfection, 1 × 10^6^ cells were plated per well in triplicate in six-well plates. The following pre-designed siRNA were used: YTHDC1 (SASI_Hs01_00062766), YTHDF3 (SASI_Hs01_00202277), YTHDC2 (SASI_Hs01_00161045), and HNRNPL (SASI_Hs01_00211076). The indicated cells were transfected with Lipofectamine 300 with 120 pmol of the indicated siRNA and 2 μg pcDNA3.1(−) Flag-circE7 expression plasmid. After 24 h, the cells were harvested for RNA as previously described. For western blots, cells were lysed in 200 μL lysis buffer with protease/phosphatase inhibitor (CST). Samples were heated at 80°C for 10 min prior to western blot.

### BaseScope and quantification

The BaseScope Reagent Kit (catalog no. 323910; Advanced Cell Diagnostics, Inc., Newark, CA, USA) was used to manually perform the BaseScope assay on FFPE tissue sections of tumors and controls per manufacturer guidelines. Custom RNA probes (Advanced Cell Diagnostics, Inc.) were designed to target fusion transcript-specific exon junctions of interest (BaseScope Probes, BA-V-HPV16-circE7, catalog no. 1195271-C1; BA-V-HPV16-E6I, catalog no. 725879-C2). Unstained FFPE 5-μm-thick tissue sections were baked in a HybEZ (Advanced Cell Diagnostics, Inc.) oven at 60°C for 1 h, followed by a series of deparaffinization steps. The sections were then left to dry at 60°C for 5 min. Once dry, RNAscope Hydrogen Peroxide (Advanced Cell Diagnostics, Inc.) was applied to each slide at room temperature and left to incubate for 10 min. The slides were then washed in distilled water twice. This step was followed by manual target retrieval in which 700 mL of 1× RNAscope Target Retrieval Reagent was poured into a beaker on a hot plate. Once the solution was warmed to 100°C, the slides were left to incubate in solution for 15 min. The slides were quickly transferred into distilled water, washed in 100% ethanol, and left out to dry at room temperature. A hydrophobic barrier was then drawn around the tissue of interest using an ImmEdge hydrophobic barrier PAP pen (H-4000). The slides were placed inside the EZ-Batch Slide Rack (Advanced Cell Diagnostics, Inc.), where RNAscope Protease III was added to each sample. The slide rack was then inserted in the HybEZ humidity control tray and incubated in the HybEZ oven (40°C, 30 min), followed by one wash in distilled water. The probes were placed in the oven in conjunction with the slide rack to equilibrate prior to use. Excess liquid was then removed from the slides post-incubation, probe mix was added and hybridized (oven at 40°C, 2 h), and slides were washed (1× RNAscope Wash Buffer twice, 2 min) and stored overnight (room temperature, 5× saline sodium citrate [SSC] buffer). The following day, a series of eight amplification steps was performed per the manufacturer’s guidelines. RED working solution (1:60, BaseScope Fast Red-B to Fast Red-A) was applied (incubation for 10 min, room temperature), and slides were counterstained using 50% Gill’s Hematoxylin I staining solution (catalog no. HXGHE1PT; American MasterTech Scientific) (room temperature, 2 min). Samples were then moved to a staining dish containing tap water (repeated until the slides were clear and sections remained purple), dipped in 0.02% ammonia water (221228; Sigma-Aldrich), washed with tap water, left out to dry (60°C, 15 min), and mounted using VectaMount (64742-48-9; Vector Laboratories). Technical controls were performed on the indicated FFPE sections and cell pellets using a human-specific housekeeping gene-positive control probe, a non-specific bacterial control probe (BaseScope Probes, BA-Hs-PPIB-1zz, Homo sapiens peptidylprolyl isomerase B [cyclophilin B] [PPIB] mRNA, catalog no. 710171; BaseScope Duplex Negative Control Probe, DapB-1ZZ, catalog no. 700141). Adjacent benign tissue was used as a negative internal control.

### Keratinocyte differentiation assay

A synthetic cDNA gBlock fragment (IDT) containing the mutant m^6^A motif (AgeI_m6A_Mut2) was cloned into the genomic HPV16 plasmid, pHPV16ANsL (gift from CM Chiang lab) using *Age*I restriction sites. Positive clones (called Mut2) were confirmed by Sanger Sequencing. Primary keratinocytes were transfected with HPV16 genomes, as previously described (47). Briefly, early-passage primary keratinocytes were co-transfected with an equal DNA ratio of either pHPV16ANsL WT or Mut2 and pCAGGS-NLS-Cre plasmids (gift from CM Chiang) using TransIT Keratinocyte Transfection Reagent (MirusBio, MIR 2800) according to the manufacturer’s protocol. Cells were selected with G418 for 6 days and maintained in keratinocyte media until the cultures reached confluence. Cells were then selected in DMEM + 10% BCS for 6 weeks to induce terminal differentiation. After 6 weeks of selection, colonies were stained overnight with 0.1% crystal violet in 50% methanol. Colonies were quantified using the ColonyArea ImageJ plugin.

### HIRT isolation

Cells (>1 × 10^6^) were scraped into PBS and washed twice in PBS before snap freezing. Cells were lysed in 200 µL Hirt lysis buffer (10 mM Tris-HCl-pH 8.0, 10 mM EDTA, 0.6% SDS; stored at RT) with gentle shaking for 10 min at room temperature. After the addition of 50 µL of 5 M NaCl, tubes were gently inverted and incubated overnight at 4°C. Tubes were centrifuged at full speed for 30 min at 4°C. The supernatant was collected and treated with RNase A (0.2 mg/mL) for 1 h at 37°C; 1,250 µL of NTB (Qiagen) was added and mixed, and DNA was purified using a spin column (Macherey-Nagel, PCR and Gel purification Kit) and eluted in 30 µL of elution buffer. The DNA (~0.5 µL) was digested with 10 units (1 µL) of *Dpn*I for 16 h, which was inactivated at 80°C for 20 min. A standard curve using 10-fold serially diluted purified plasmids was used to establish a standard curve, and qPCR was run using the DpnI-digested samples.

### Exonuclease V integration assay

PCR-based analysis of viral genome status was performed using methods described by Myers et al. (48). Briefly, 20 ng of genomic DNA was either treated with exonuclease V (RecBCD, NEB) in a total volume of 30 µL or left untreated for 1 h at 37°C, followed by heat inactivation at 95°C for 10 min; 2 ng of digested/undigested DNA was then quantified by real-time PCR using a 7500 FAST Applied Biosystems thermocycler with SYBR Green PCR Master Mix (Applied Biosystems) and 100 nM of primer in a 20 μL reaction. Nuclease-free water was used in place of the template for a negative control. The following cycling conditions were used: 50°C for 2 min, 95°C for 10 min, 40 cycles at 95°C for 15 s, and a dissociation stage of 95°C for 15 s, 60°C for 1 min, 95°C for 15 s, and 60°C for 15 s. Separate PCR reactions were performed to amplify regions of interest. Episomal status was calculated by dCt(Exo-no Enzyme), followed by comparison of dCt(HPV gene) with dCt(mitochondrial DNA) and dCt(GAPDH).

## Data Availability

The data that support the findings of this study are available from the corresponding author upon request.

## SUPPLEMENTAL MATERIAL

Supplemental material jvi.01284-26-s0001.pdf Tables S1 and S2; Fig. S1 to S9.

ASM does not own the copyrights to Supplemental Material that may be linked to, or accessed through, an article. The authors have granted ASM a non-exclusive, world-wide license to publish the Supplemental Material files. Please contact the corresponding author directly for reuse.
