# Supplementary material for: Regulation and function of the HPV16 CircE7 RNA
Source: J Virol. 2026 Sep 17;100(10):e01284-26. doi: 10.1128/jvi.01284-26 (PMC13712926; doi:10.1128/jvi.01284-26)
Supplement: Supplementary file 1 [file jvi.01284-26-s0001.pdf]

| **Supplemental Table 1 – Synthetic DNA sequences** |
|---|
| **AgeI_m6A_mut2 (for mutagenesis of pHPV16ANsL plasmid)**<br>**ACTAAGGGCGTAACCGAAATCGGTTGAACCGAAACCGGTTAGTATAAAAGCAGACATTTTATGCA**<br>**CCAAAAGAGAACTGCAATGTTTCAGGACCCACAGGAGCGACCCGGAAAGTTACCACAGTTATGC**<br>**ACAGAGCTGCAAACAACTATACATGATATAATATTAGAATGTGTGTACTGCAAGCAACAGTTACTG**<br>**CGACGTGAGGTATATGACTTTGCTTTTCGGGATTTATGCATAGTATATAGAGATGGGAATCCATATG**<br>**CTGTATGTGATAAATGTTTAAAGTTTTATTCTAAAATTAGTGAGTATAGACATTATTGTTATAGTGTGT**<br>**ATGGAACAACATTAGAACAGCAATACAACAAACCGTTGTGTGATTTGTTAATTAGGTGTATCAATT**<br>**GTCAAAAGCCACTGTGTCCTGAAGAAAAGCAAAGACATCTGGACAAAAAGCAAAGATTCCATAA**<br>**TATAAGGGGTCGGTGGACCGGTCGATGTATGTCTTGTTGCAGATCATCAAGAACACGTAGAG** |
| **16ER_splice_Not_Bam (for generation of splice reporter plasmid)**<br>**TCGAGCGGCCGCCACTGCAATGTTTCAGGACCCACAGGAGCGACCCAGAAAGTTACCACAGTT**<br>**ATGCACAGAGCTGCAAACAACTATACATGATATAATATTAGAATGTGTGTACTGCAAGCAACAGTT**<br>**ACTGCGACGTGAGGTATATGACTTTGCTTTTCGGGATTTATGCATAGTATATAGAGATGGGAATCC**<br>**ATATGCTGTATGTGATAAATGTTTAAAGTTTTATTCTAAAATTAGTGAGTATAGACATTATTGTTATAG**<br>**TTTGTATGGAACAACATTAGAACAGCAATACAACAAACCGTTGTGTGATTTGTTAATTAGGTGTATT**<br>**AACTGTCAAAAGCCACTGTGTCCTGAAGAAAAGCAAAGACATCTGGACAAAAAGCAAAGATTCC**<br>**ATAATATAAGGGGTCGGTGGACCGGTCGATGTATGTCTTGTTGCAGATCATCAAGAACACGTAGA**<br>**GAAACCCAGCTGTAATCATGCATGGAGATACACCTACATTGCATGAATATATGTTAGATTTGCAAC**<br>**CAGAGACAACTGATCTCTACTGTTATGAGCAATTAAATGACAGCTCAGAGGAGGAGGATGAAATA**<br>**GATGGTCCAGCTGGACAAGCAGAACCGGACAGAGCCCATTACAATATTGTAACCTTTTGTTGCAA**<br>**GTGTGACTCTACGCTTCGGTTGTGCGTACAAAGCACACACGTAGACATTCGTACTTTGGAAGACC**<br>**TGTTAATGGGCACACTAGGAATTGTGTGCCCCATCTGTTCTCAGAAACCATAAGGATCCGAGCT** |
| **16ER_silence_mut_Age_Bam (for mutagenesis of splice reporter plasmid)**<br>**TCGGTGGACCGGTCGATGTATGTCTTGTTGCAGATCATCAAGAACACGTAGAGAAACCCAGCTG**<br>**TAATCATGCATGGAGATACACCTACATTGCATGAATAcATGcTcGccTTGCAACCAGAGACAACTGA**<br>**TCTCTACTGTTATGAGCAATTAAATGACAGCTCAGAGGAGGAGGATGAAATAGATGGTCCAGCTG**<br>**GACAAGCAGAACCGGACAGAGCCCATTACAATATTGTAACCTTTTGTTGCAAGTGTGACTCTACG**<br>**CTTCGGTTGTGCGTACAAAGCACACACGTAGACATTCGTACTTTGGAAGACCTGTTAATGGGCAC**<br>**ACTAGGAATTGTGTGCCCCATCTGTTCTCAGAAACCATAAGGATCCGAGCT** |
| **16ER_m6Amut_Not_Age (for mutagenesis of splice reporter plasmid)**<br>**CGAGCGGCCGCCACTGCAATGTTTCAGGACCCACAGGAGCGACCCAGAAAGTTACCACAGTTA**<br>**TGCACAGAGCTGCAAACAACTATACATGATATAATATTAGAATGTGTGTACTGCAAGCAACAGTTA**<br>**CTGCGACGTGAGGTATATGACTTTGCTTTTCGGGATTTATGCATAGTATATAGAGATGGGAATCCAT**<br>**ATGCTGTATGTGATAAATGTTTAAAGTTTTATTCTAAAATTAGTGAGTATAGACATTATTGTTATAGTT**<br>**TGTATGGAACAACATTAGAACAGCAATACAACAAACCGTTGTGTGATTTGTTAATTAGGTGTATTAt**<br>**CTGTCAAAAGCCACTGTGTCCTGAAGAAAAGCAAAGACATCTGGACAAAAAGCAAAGATTCCAT**<br>**AATATAAGGGGTCGGTGGACCGGTCGATGTATGTC** |

| Supplemental Table 2 – Primer sequences | | |
|---|---|---|
| **Name** | **Sequence (5'-3')** | **Function** |
| **HPV16_circE7_7861_F** | ACTAGGAATTGTGTGCCCCA | HPV16 circE7 |
| **HPV16_circE7_7544_R** | GGTCCACCGACCCCTTATATT | |
| **HPV16_linear_E6/E7_7410_F** | GAACAGCAATACAACAAACCGT | Linear E6 |
| **HPV16_linear_E6/E7_7708_R** | CTCCTCCTCTGAGCTGTCATT | |
| HPV16_full_length_E6/E7_set1_F | GTGTACTGCAAGCAACAGTTA | Linear E6 |
| HPV16_full_length_E6/E7_set1_R | CCCATCTCTATATACTATGCATAAATCC | |
| **HPV16_E6*I_7259_F** | GCGACGTGAGGTGTATTAAC | E6*I specific primer (WT) |
| **HPV16_E6*I_7758_R** | TAATGGGCTCTGTCCGGTTC | |
| **HPV16_E6*I_Mut2_F** | TACTGCGACGTGAGGTGTAT | E6*I specific primer (Mut2) |
| **HPV16_E6*I_Mut2_R** | ATGTAGGTGTATCTCCATGC | |
| **CircE7_IRESmut_F** | TGCACTTATCATGCATGGAGATACAC | IRES motif mutagenesis |
| **CircE7_IRESmut_R** | CCCAAAGTCTACGTGTTCTTGATGATC | |
| **circE7_m6A_mut_F** | AGGGCGTAACCGAAATCG | Mut2 cloning |
| **circE7_m6A_mut2_R** | CGTGTTCTTGATGATCTGC | |
| **Actin_F** | CAGCCATGTACGTTGCTATCCA | qPCR control |
| **Actin_R** | TCACCGGAGTCCATCACGAT | |
| **Circ_HIPK3_F** | CAACATATCTACAATCTCGGTAC | Polysome PCR control |
| **Circ_HIPK3_R** | ACAACTGCTTGGCTCTACT | |
| ExoV HPV16 E6 F | TTGCTTTTCGGGATTTATGC | ExoV assay |
| ExoV HPV16 E6 R | CAGGACACAGTGGCTTTTGA | |
| ExoV HPV16 E2 F | TGGAAGTGCAGTTTGATGGA | ExoV assay |
| ExoV HPV16 E2 R | CCGCATGAACTTCCCATACT | |
| ExoV mitochondrial DNA F | CAGGAGTAGGAGAGAGGGAGGTAAG | ExoV control |
| ExoV mitochondrial DNA R | TACCCATCATAATCGGAGGCTTTGG | |
| ExoV GAPDH DNA F | GGAGCGAGATCCCTCCAAAAT | ExoV control |
| ExoV GAPDH DNA R | GGCTGTTGTCATACTTCTCATGG | |

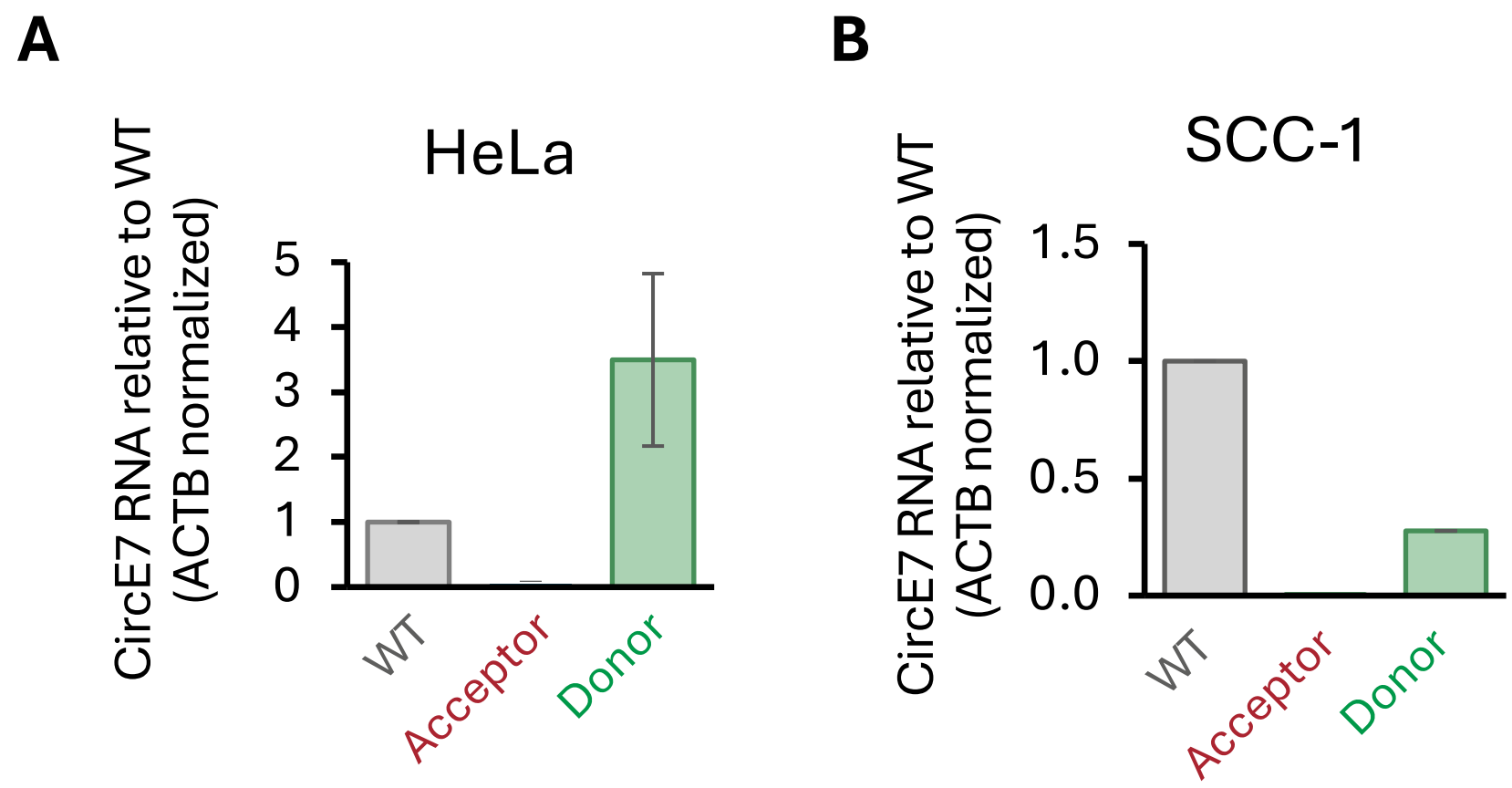


**Supplemental Figure 1. Mutating m$^6$A reduces circE7 in multiple cell lines.** CircE7 RNA assess by RT-qPCR in **(A)** HeLa and **(B)** SCC-1 cells transfected with wild type (WT), m6A acceptor mutant (Acceptor), and m6A donor mutant (Donor).

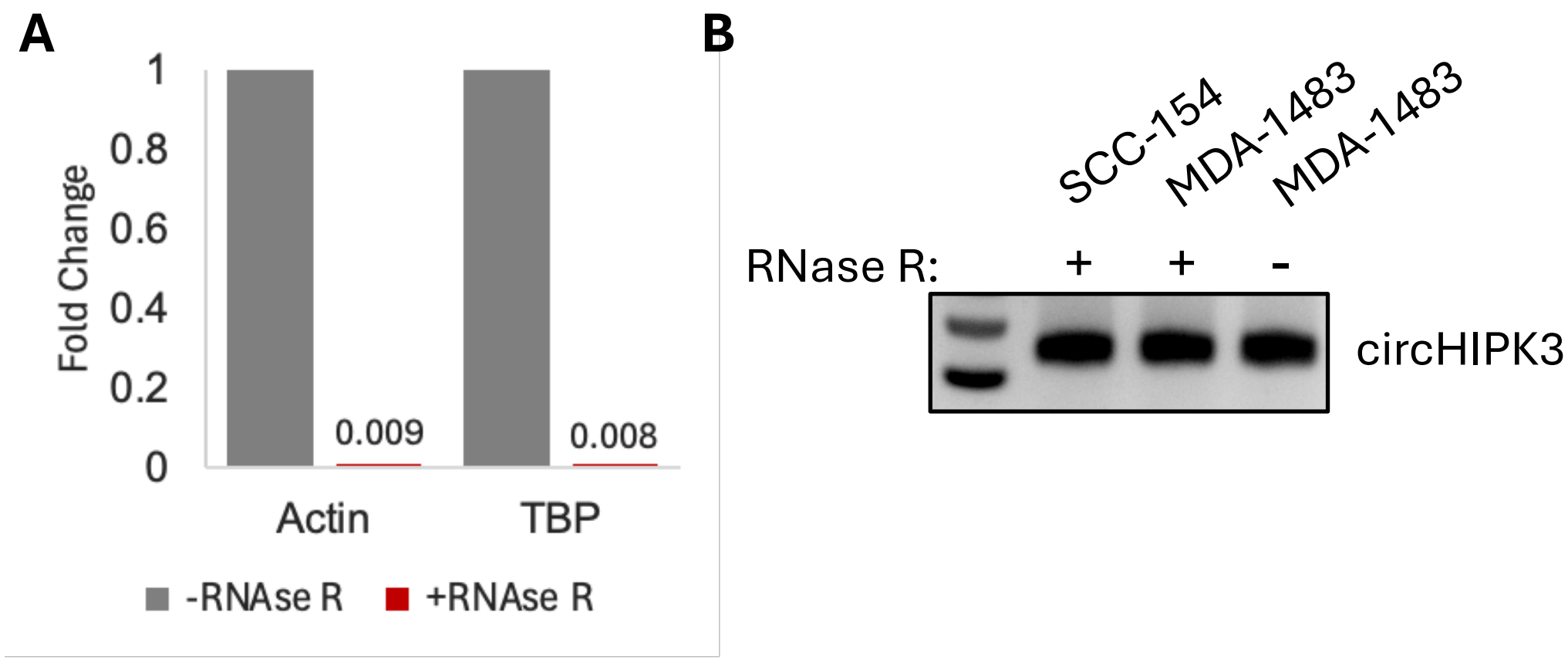


**Supplemental Figure 2. Confirmation of RNase R digestion of linear RNAs. (A)** RT-qPCR of linear RNAs in MDA-1483 polysome associated RNA fractions with or without RNase R treatment. **(B)** Endpoint PCR of circHIPK3 in the same samples.

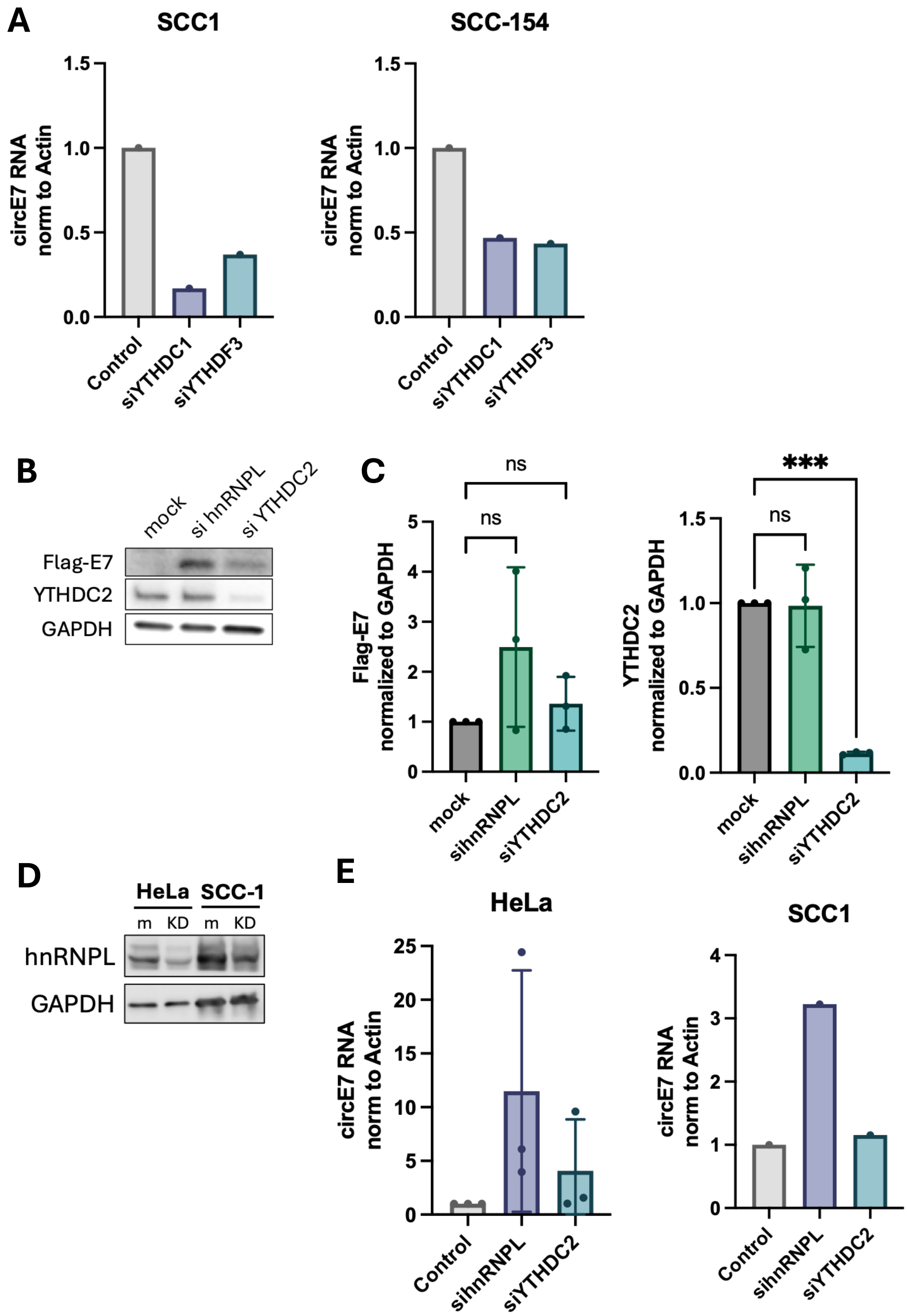


**Supplemental Figure 3. Additional m$^6$A readers repress circE7.**
**(A)** RT-qPCR of circE7 after knockdown of various YTHDC1 or YTHDF3 m$^6$A readers in SCC1 and SCC-154 cell lines. n=1. **(B)** Representative western blot of Flag-tagged E7 protein, YTHDC2 and GAPDH proteins after mock, si-hnRNPL, or siYTHDC2 transfection in 293T cells. **(C)** Quantification of Flag-E7 (left) and YTHDC2 (right) western blots. One way ANOVA. ***p<0.001. **(D)** Western blot showing hnRNPL KD in HeLa and SCC1 cells **(E)** RT-qPCR of circE7 RNA after knockdown of various proteins. Normalized to Actin RNA then control condition. For the HeLa control, si- hnRNPL, and siYTHDC2 n=3; all others n=1. Conditions are not statistically significant.

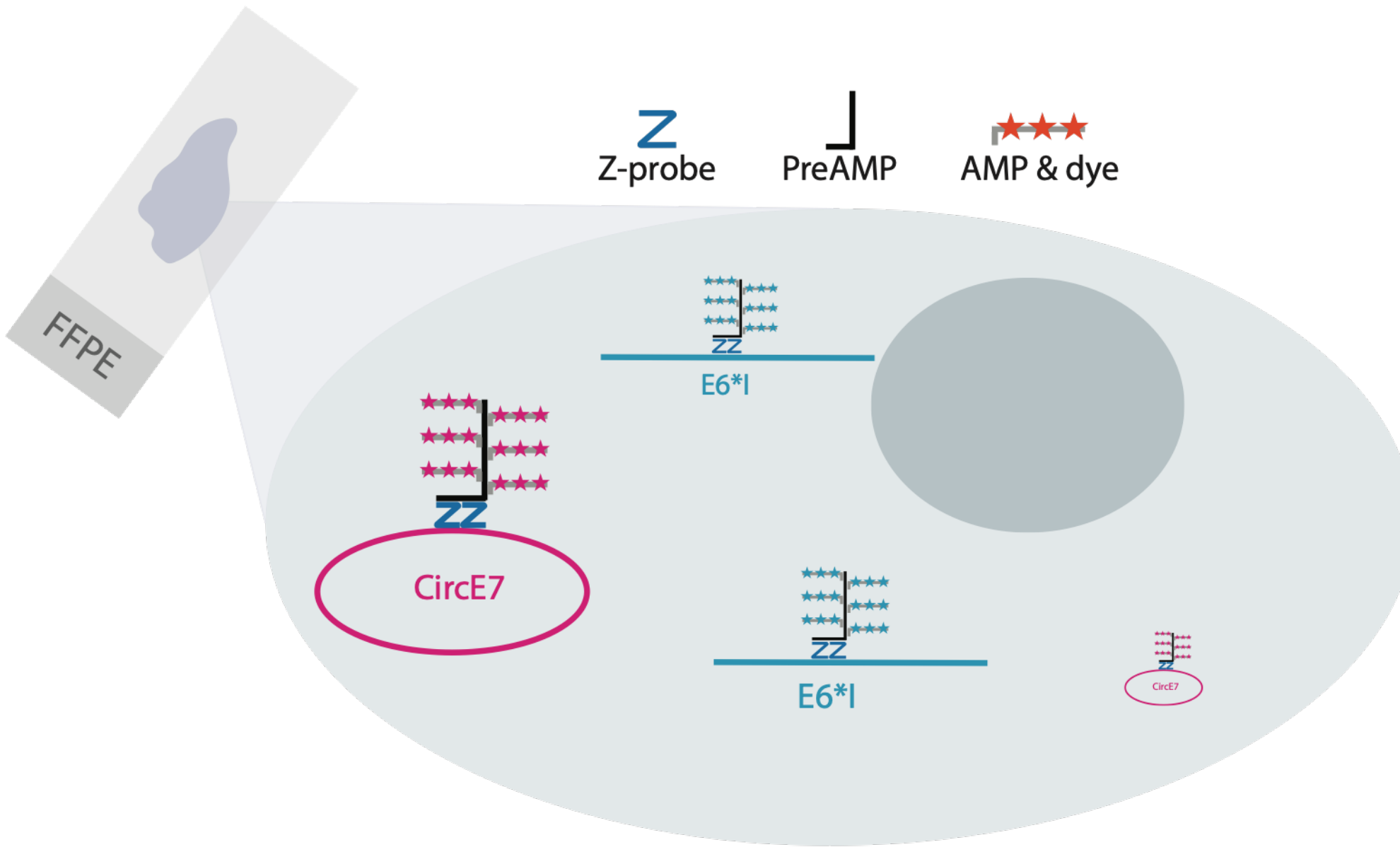


**Supplemental Figure 4. BaseScope schematic**
BaseScope RNA-ISH detection of RNA. Double “Z-probes” allow for discrimination of circE7 and E6*I splice isoforms.

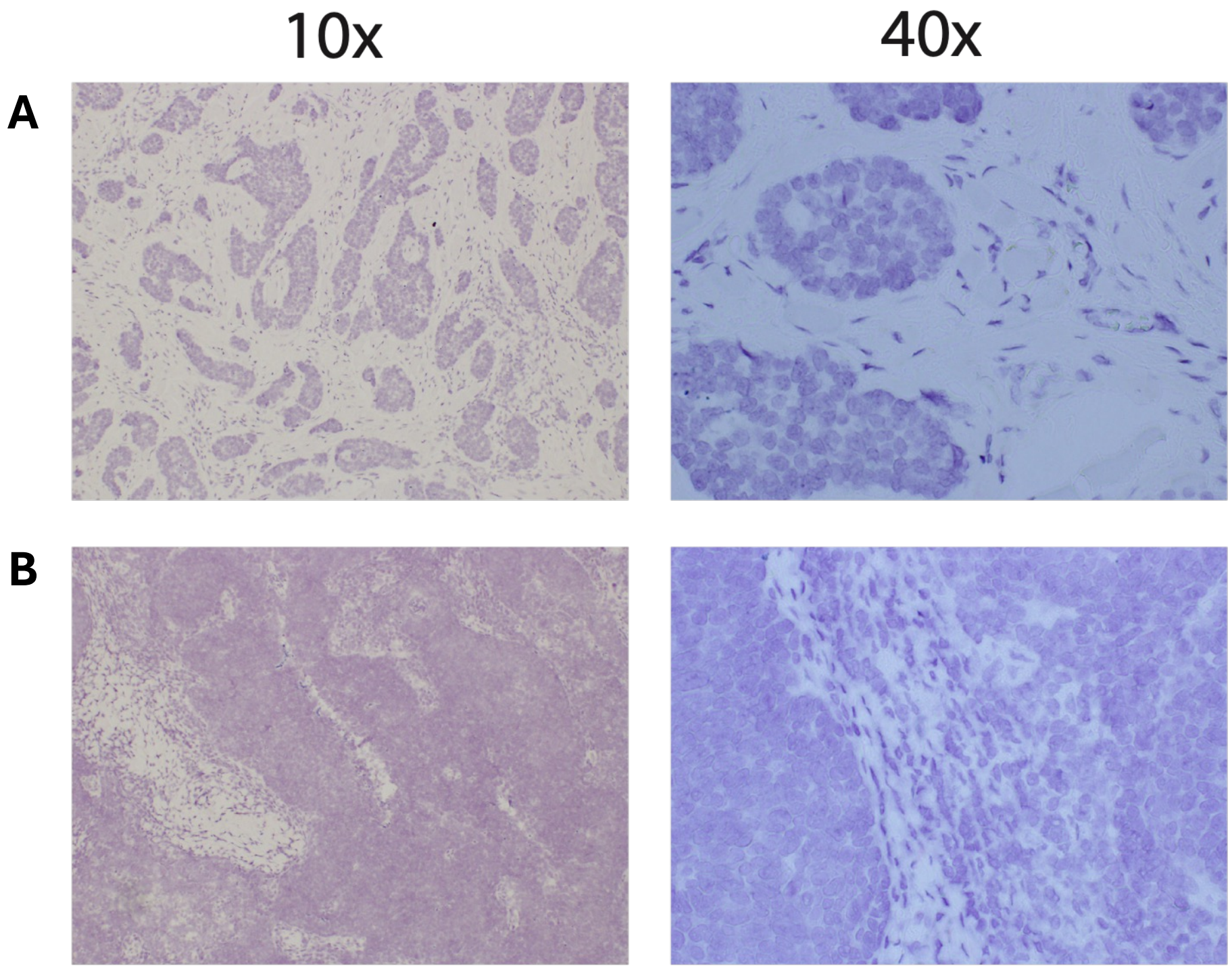


**Supplemental Figure 5. BaseScope circE7 and E6*I negative controls**. **(A)** Microsecretory adenocarcinoma, negative for HPV **(B)** Mucoepidermoid carcinoma negative for linear E6 and circE7.

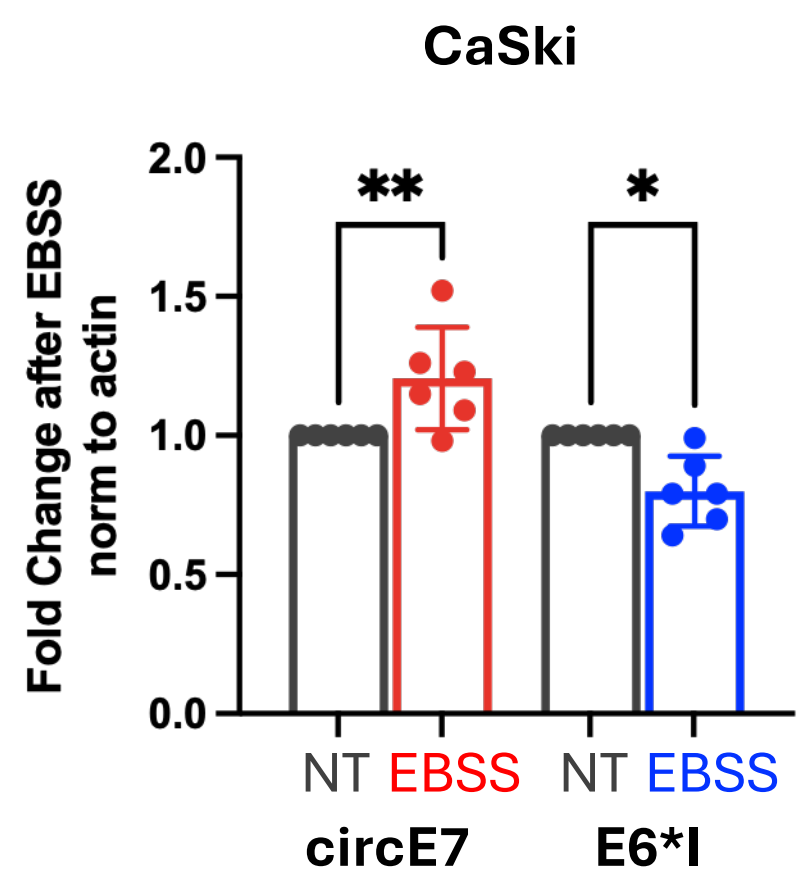


**Supplemental Figure 6. E6*I during EBSS starvation.**
Fold change of HPV RNAs after EBSS starvation in CaSki cells assess by RT-qPCR. Normalized to actin and control growth condition. **p<0.01, *p<0.05, t-test.

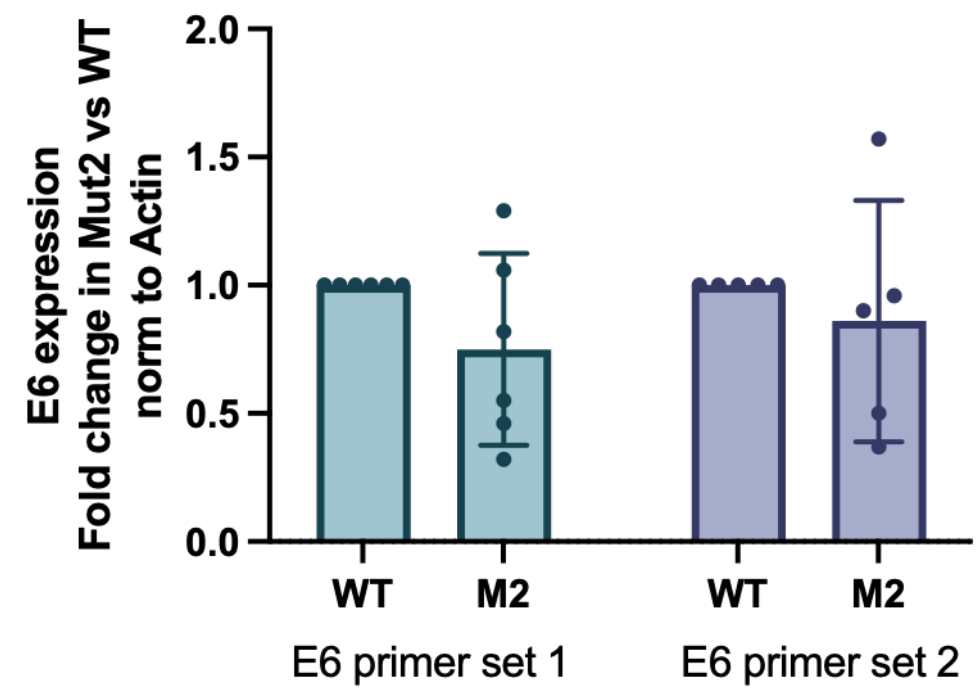


**Supplemental Figure 7. Linear E6 transcript in WT and Mut2 pHPV genomes.** Relative expression of E6-E7 RNA that encodes full length E6 protein quantified by RT-qPCR. Change between WT and M2 is not statistically significant.

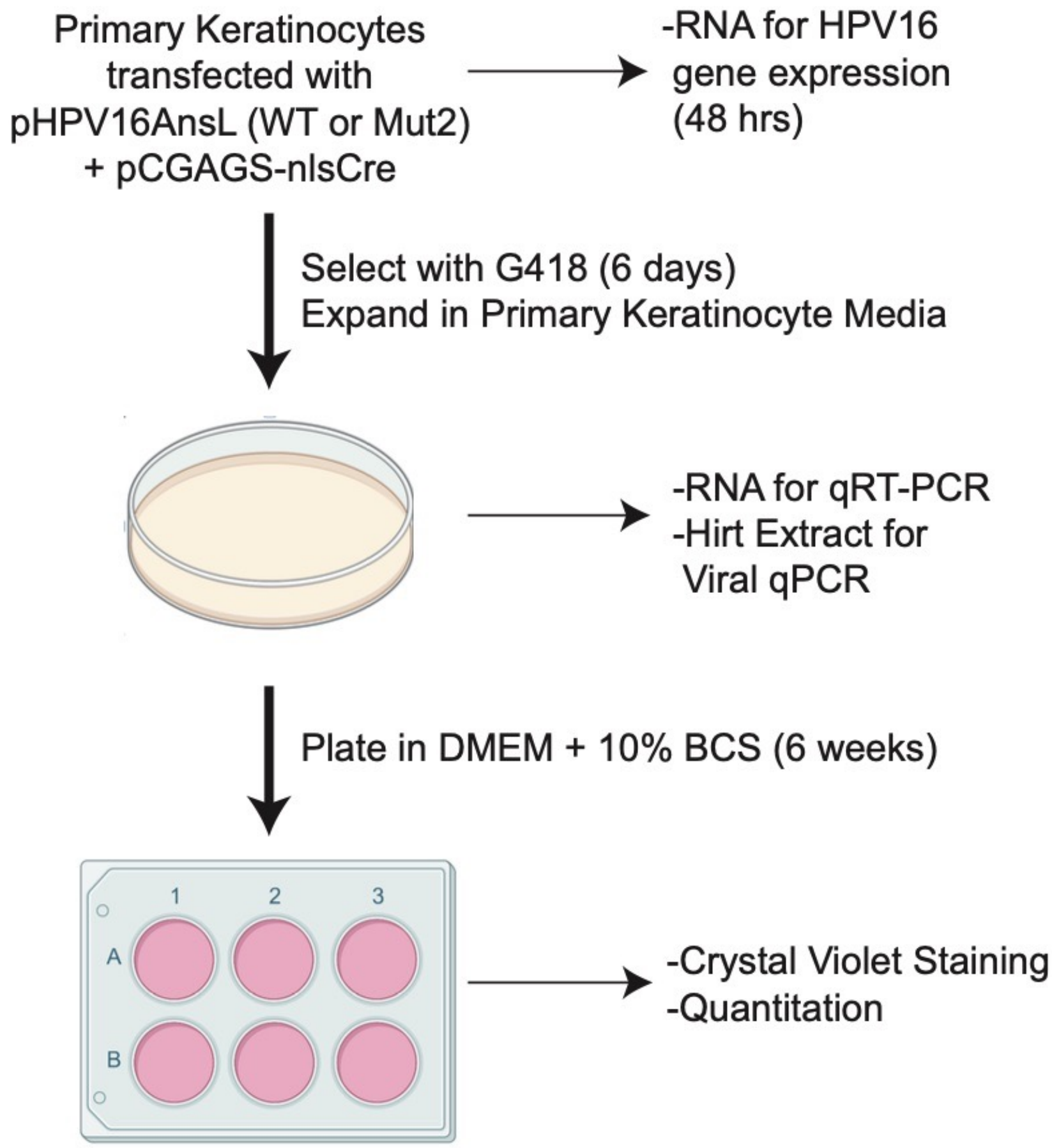


**Supplemental Figure 8. Experimental design for HPV16 infection evaluation.**

Primary keratinocytes were grown in primary keratinocyte media and co-transfected with the pHPV16AnsL genome, either wild type or Mut2, and pCGAGS-nlsCre. 48 hours after transfection, RNA was harvested from transfected keratinocytes for quantifying HPV16 gene expression. Cells were selected with G418 for 6 days and expanded in primary keratinocyte media. After selection, cells were harvested for RNA and Hirt extracts. Selected keratinocytes were then grown in DMEM with 10% bovine calf serum for up to 6 weeks and monitored at various timepoints via crystal violet staining.

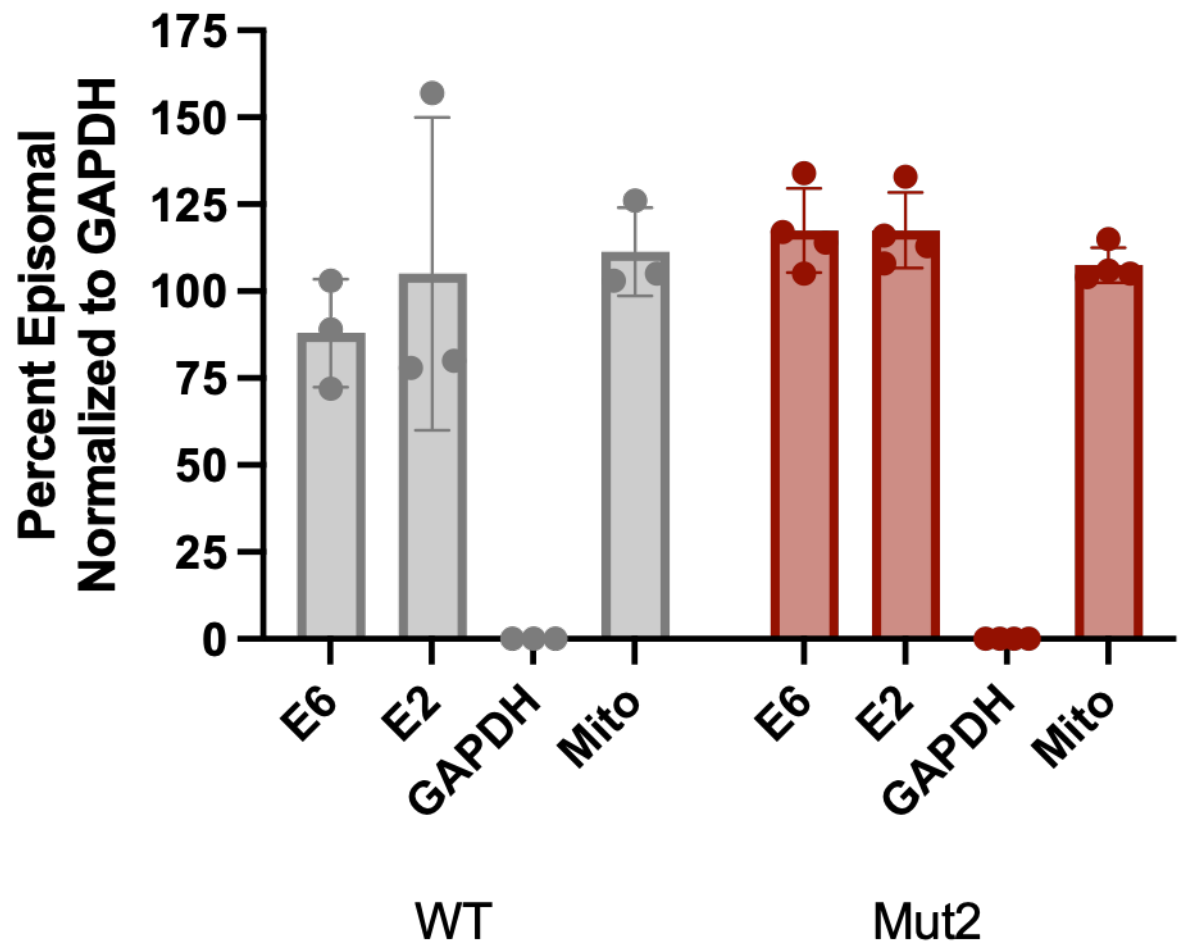


**Supplemental Figure 9. Both WT and Mut2 HPV genomes remains episomal in primary keratinocytes.** Percent genome integration of HPV wild type (WT) and mut2 genomes was determined by ExoV digestion. Primary keratinocytes were transfected with HPV genomes, selected with G418 and harvested for total genomic DNA. There is no statistical difference between WT and Mut2 for any of the targets (t-test).
